# How Good Are RGB Cameras Retrieving Colors of Natural Scenes and Paintings?—A Study Based on Hyperspectral Imaging

**DOI:** 10.3390/s20216242

**Published:** 2020-11-01

**Authors:** João M. M. Linhares, José A. R. Monteiro, Ana Bailão, Liliana Cardeira, Taisei Kondo, Shigeki Nakauchi, Marcello Picollo, Costanza Cucci, Andrea Casini, Lorenzo Stefani, Sérgio Miguel Cardoso Nascimento

**Affiliations:** 1 Centre of Physics, Gualtar Campus, University of Minho, 4710-057 Braga, Portugal; alexmonteiro1995@gmail.com (J.A.R.M.); smcn@fisica.uminho.pt (S.M.C.N.); 2 Faculty of Fine Arts, University of Lisbon, 1649-004 Lisboa, Portugal; ana.bailao@gmail.com (A.B.); lilianacardeira@gmail.com (L.C.); 3 Research Center for Science and Technology of the Arts—Portuguese Catholic University, Centre Regional of Porto, 4169-005 Porto, Portugal; 4 Toyohashi University of Technology, Aichi 441-8580, Japan; kondo15@vpac.cs.tut.ac.jp (T.K.); nakauchi@tut.jp (S.N.); 5 Istituto di Fisica Applicata “Nello Carrara” del Consiglio Nazionale delle Ricerche (IFAC-CNR), Via Madonna del piano 10, 50019 Firenze, Italy; m.picollo@ifac.cnr.it (M.P.); c.cucci@ifac.cnr.it (C.C.); a.casini@ifac.cnr.it (A.C.); l.stefani@ifac.cnr.it (L.S.)

**Keywords:** hyperspectral imaging, natural scenes, paintings, chromatic errors, color difference, number of colors.

## Abstract

RGB digital cameras (RGB) compress the spectral information into a trichromatic system capable of approximately representing the actual colors of objects. Although RGB digital cameras follow the same compression philosophy as the human eye (OBS), the spectral sensitivity is different. To what extent they provide the same chromatic experiences is still an open question, especially with complex images. We addressed this question by comparing the actual colors derived from spectral imaging with those obtained with RGB cameras. The data from hyperspectral imaging of 50 natural scenes and 89 paintings was used to estimate the chromatic differences between OBS and RGB. The corresponding color errors were estimated and analyzed in the color spaces CIELAB (using the color difference formulas ΔE^*^_ab_ and CIEDE2000), *J_z_a_z_b_z_*, and iCAM06. In CIELAB the most frequent error (using ΔE^*^_ab_) found was 5 for both paintings and natural scenes, a similarity that held for the other spaces tested. In addition, the distribution of errors across the color space shows that the errors are small in the achromatic region and increase with saturation. Overall, the results indicate that the chromatic errors estimated are close to the acceptance error and therefore RGB digital cameras are able to produce quite realistic colors of complex scenarios.

## 1. Introduction

Digital color cameras acquire images by sampling the spatial and spectral information available, much like the human eye does [1]. In what concerns color, the acquisition process compresses the spectral information reflected from the objects into three components, discarding most of the spectral information initially available. The entire process produces colors that are device-dependent, different from camera to camera, and do not map linearly to the device-independent tristimulus values, which represent human visual perception [2]. Thus, cameras produce colors that are an approximation of what we see when looking at those scenarios. On the other hand, hyperspectral images acquire the spatial information without the chromatic compression found in digital cameras, maintaining the spectral properties of the light signal, information relevant to research in vision [3]. 

It is possible to improve the fidelity of digital cameras computationally using training data, but the efficacy of the training set is very dependent on the type of images to be acquired [2,4] or the camera settings [5]. If the colors are close to the neutral region, the differences are small [6], but otherwise are large [7,8]. On the other hand, some of the colors may be made metameric by the process [9]. Another way is to calibrate the spectral response of a digital camera using interference filters to improve the accuracy of the color recorded [10]. It is also possible to increase the number of spectral filters, or bands, to be registered, making the image acquired multispectral [11], enabling partial spectral reconstruction of the spectral reflectance of the objects imaged [12]. Even in the specific case of fixed illumination and precise camera calibration, it is still not possible to obtain a perfect reproduction of the colors, although considerable improvements are obtained [13]. Studies based on optimal color stimuli, i.e. optimal colors, show that the gamut of colors for a standard observer is significantly larger than the gamut provided by a digital camera [14,15,16]. 

Typical digital cameras, however, are trichromatic, registering only three bands in the red, green, and blue regions of the light spectrum, and natural images do not have optimal colors but a very specific color distribution and statistics [17,18]. To what extent typical cameras are faithful to the real colors of complex natural scenarios? Although much work has been done characterizing fidelity with simple stimuli [6,7,8,14,15,16] it is unclear how they perform with complex imagery, such as complex natural scenes. 

We address this question by using hyperspectral images of natural scenes and paintings, which contains the contexts and information required for the analysis presented.The spectral reflectance data in the hyperspectral images was converted into colors as perceived by a human observer (OBS) and by a typical digital camera (RGB). The colors obtained for the OBS and RGB were then compared, the chromatic errors estimated, and the frequency of the errors computed. The color difference formulas CIEDE and CIEDE2000 were used in the CIELAB color space and the Euclidean distance formula was used in the *J_z_a_z_b_z_* and iCAM06 color spaces to compute the color differences. 190 × 10^6^ pixels were used, spanning from natural colors to colors presented in paintings from Portugal, Italy, and Japan. 

## 2. Materials and Methods

**Figure 1:**
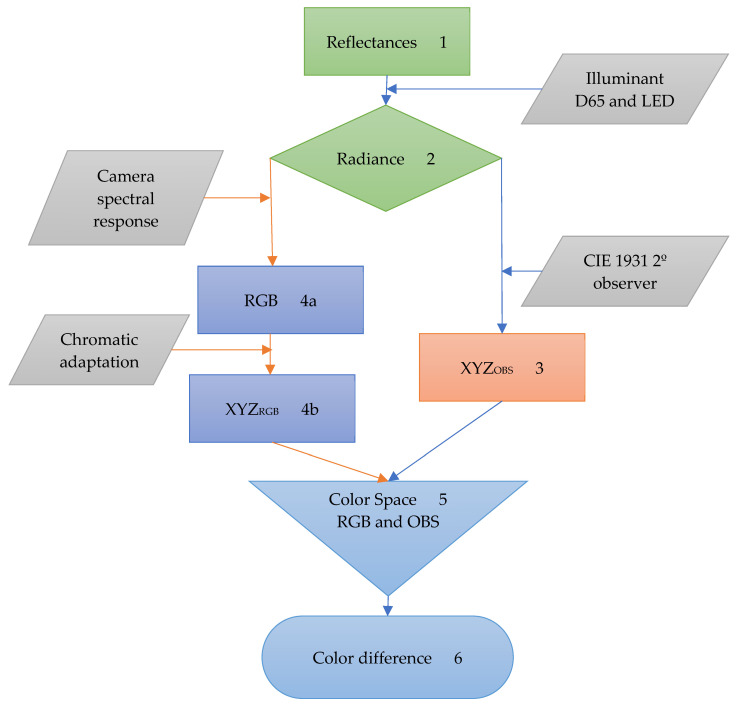
Workflow used to estimate the color differences between the human eye and a RGB digital camera. From a set of reflectance functions derived from spectral imaging (1), the colors processed by the digital camera (4, orange lines) and those seen by the standard observer (3, blue lines) were estimated and compared in the same color space (5 and 6).

Spectral imaging data of paintings and natural scenes acquired using several hyperspectral systems (HIS) were converted into a color space. The color coordinates were computed assuming the conversion of the spectral data of each image pixel into a color coordinate for the CIE 1931 2º Standard Observer [19] and a commercial digital RGB camera [10]. The color difference between the estimated colors for both devices was then estimated and the frequency of error computed. Figure 1 represents the workflow used to estimate the color differences. 

Starting with the spectral data for each image pixel, in the form of reflectances (step 1), the radiance (step 2) was estimated for the CIE D65 illuminant [18] and an LED with a CCT of 3176ºK, close to the tungsten or halogen light sources, traditionally considered the default museum illumination [20,21,22,23].

The radiance data was then processed into tristimulus values to estimate the XYZ_OBS_ (step3) and the XYZ_RGB_ (step 4). To estimate the XYZ_OBS_ values, the CIE 1931 2º observer was considered [19]. This allows the estimation of the real colors. To estimate the XYZ_RGB_ values, the spectral sensitivity of an RGB digital camera (Kodak KAF-10500 image sensor presented in a Leica M8, Leica Camera INC. Allendale, NJ 07401, USA) was used, as described elsewhere [10]. This step (step 4a) returns the RGB chromaticity coordinates at the camera level. To convert these coordinates from the camera level to the observer level [14,15,16], a linear transformation was used to convert the RGB to XYZ_RGB_ for data assuming D65 (step 4b—*rgb2xyz.m*, MATLAB, MathWorks, Natick, MA, USA). For the LED we assume the same transformation but introduced a Bradford chromatic adaptation [24,25] (this chromatic adaptation was used as it performs better than the von Kries chromatic adaptation, as described elsewhere [25]). This allows the estimation of the colors as if they were displayed in a typical display monitor. Both tristimulus values were then converted into a color space with associated color difference formula (step 5) [19,26,27,28,29]. The difference between the colors obtained from the RGB camera and the CIE observer was then computed and the frequency of errors estimated (step 6). 

When converting the spectral reflectance to the CIELAB color coordinates, some of the pixel colors could not be converted properly due to noise in the data. Nevertheless, only 2.5% of the total of the number of pixels used were not considered and were removed from the analysis.

### 2.1. Paintings and Natural Scenes

**Figure 2:**
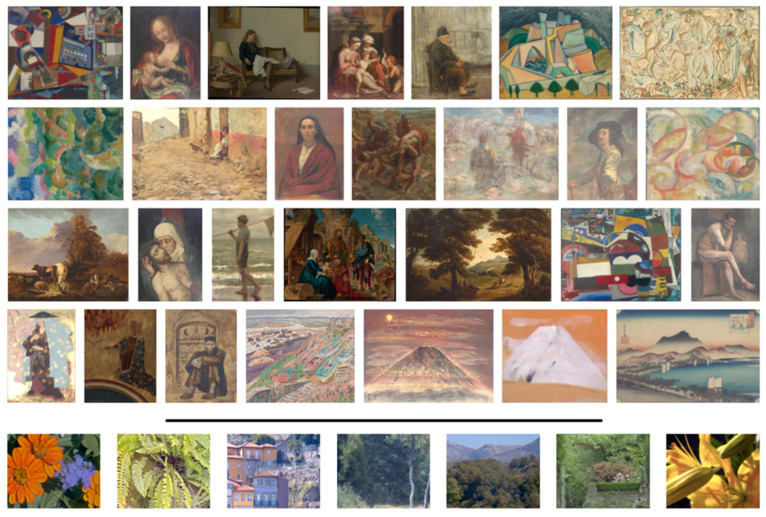
Representative images of the 89 paintings (above line) and 50 natural scenes (bellow line) analyzed in this work.

Figure 2 represents some examples of the images used in this work. Images were derived from hyperspectral imaging assuming the D65 illuminant. Above the line are represented samples of the 89 paintings analyzed. Paintings from Portugal, Japan, and Italy were used. Below the line are represented samples of the 50 natural scenes analyzed, all acquired in the north of Portugal. The final set composed of 139 images provided 197,936,935 pixels for analysis. 

All images were acquired using hyperspectral imaging and delivered in each image pixel the reflectance spectra for the area imaged. The reflectance spectra were from 400 nm to 720 nm in 10 nm steps. Only in the case of the Japanese paintings, the reflectance spectra data was available from 420 to 720 nm in 10 nm steps. 

The reflectance spectra of the Portuguese paintings and the natural scenes were acquired using a spatial resolution of 1024 × 1344 pixels. The acquisition procedure and the accuracy of the methodology was described elsewhere [3,30], but the accuracy of the system in retrieving the spectral profile is within 2%, with an average color difference error of about 2.2 in the CIELAB color space. 

The reflectance spectra of the Japanese paintings were acquired using a spatial resolution of 1024 × 1932 pixels (Nuance-VIS, Cambridge Research and Instrumentation, Inc., Hopkinton, MA, USA). The reduced bandwidth from 400 to 420 of these images, when compared to the other paintings from Portugal and Italy, are not expected to impact the overall result. Simulations using Portuguese paintings and comparing the same image with and without the mentioned bandwidths, returned color differences of about 0.4 (±0.6) in the CIELAB color space [31].

The Italian paintings were acquired using a line scanning over the visible and infrared spectrum, but the spectral and spatial resolution of the data used was adjusted to be coincident with the data of the other images [32,33,34]. To achieve so, a spatial sampling was done to reduce the spatial resolution and the spectral resolution was trimmed and sampled from 400 nm to 720 nm in 10 nm steps. 

### 2.2. Camera and Standard Observer Spectral Sensitivity 

**Figure 3:**
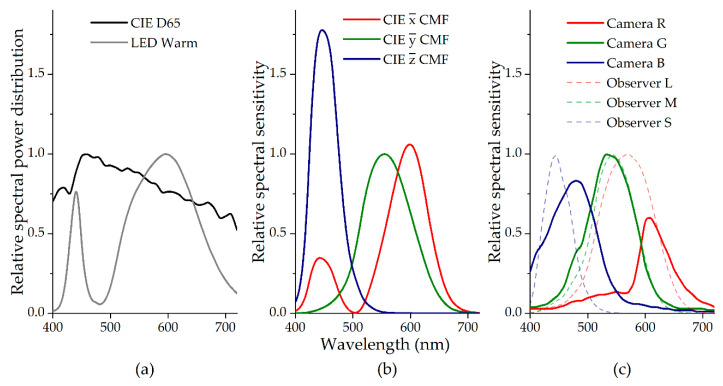
Relative spectral power distributions of the illuminants used (**a**), the CIE 1931 Observer color matching functions (**b**) and the digital camera sensor sensitivity (**c**), compared with the human cones sensitivity (dashed lines).

Figure 3 represents the relative spectral profile of the illuminants, the CIE 1931 2º Standard Observer and the digital camera RGB sensor spectral sensitivities used in the work. Panel (a) represents the CIE D65 standard illuminant [19] using a black line and the LED with a CCT of 3176ºK (LXML—PWW1-0060, Luxeon, Philips Lumileds Lighting Company, San Jose, CA, EUA) using a grey line. In both cases, the maximum was set to 1. Panel (b) represents the CIE 1931 2º Standard Observer color matching functions [19] in red, green, and blue for the $\bar{x}\left(\lambda \right),\;\bar{y}\left(\lambda \right)$ and $\bar{z}\left(\lambda \right)$ color matching function, respectively, normalized so that the maximum of $\bar{y}\left(\lambda \right)$ is 1. Panel (c) represents the digital camera spectral sensitivity of the $\bar{r}\left(\lambda \right),\;\bar{g}\left(\lambda \right)$ and $\bar{b}\left(\lambda \right)$ channel with the red, green, and blue line, respectively, normalized so that the maximum of $\bar{g}\left(\lambda \right)$ is 1. Also represented are the cone fundamentals normalized to unity, representing the l(λ), m(λ) and s(λ) cones as red, green, and blue dashed lines, respectively [35].

### 2.3. Color Differences

The CIELAB color space was used as a starting point because it is a perceptual system [19], although with some limitations regarding uniformity [29]. To estimate the chromaticity coordinates of each image pixel the tristimulus values were converted into $L^{*}$ (the lightness of the color, 0 for black and 100 for white), $a^{*}$ (with negative values representing green and positive values representing red) and $b^{*}$ (with negative values representing blue and positive values representing yellow) assuming the CIE D65 and the LED illuminants, as described elsewhere [3,19]. The CIELAB color space has an associated color difference formula $(\Delta E_{ab}^{*}=\sqrt{{\left(\Delta L^{*}\right)}^{2}+{\left(\Delta a^{*}\right)}^{2}+{\left(\Delta b^{*}\right)}^{2}}$), based on the Euclidean distance between two chromaticity coordinates [18] by comparing the difference for each color attribute. To overcome some of the non-uniformities of the space, the CIE introduced the CIEDE2000 color difference formula [19,29]. The color differences were computed using the $\left(\Delta E_{ab}^{*}\right)$ and the CIEDE2000 color difference formulas. In the case of the CIEDE2000 color difference formula, the parametric factors $k_{L},\;k_{C}$, and $k_{H}$ were set to one, the default values [36].

To estimate the color differences on a more recent and more uniform color space, the chromaticity coordinates and color differences for the *J_z_a_z_b_z_* color space [28] were computed. Its uniformity is superior to the CIELAB, but the color difference between two chromaticity coordinates is also estimated by computing the Euclidean distance between the two points. The *J_z_a_z_b_z_* color space is a simple computation color space with superior outcomes in what concerns the uniformity and estimation of small or large color differences [37].

The CIELAB and the *J_z_a_z_b_z_* color spaces are optimal for individual color comparison surrounded by a background. The stimuli analyzed here are complex spatial structures, as found in paintings and natural scenes. As such, these color spaces may not fully describe the complex pixel color differences. An image appearance model can provide a better description of the complex color stimuli that complex images encapsulate [26]. One of such models is the iCAM06 color appearance model [27]. This color appearance model takes as inputs the XYZ tristimulus values and take into account the structure of the image itself as the surrounding environment, tagged with absolute luminance to predict the degree of the chromatic adaptation and the overall image contrast, increase in perceived colorfulness and image contrast with the luminance (Hunt and Stevens effect, respectively) [38]. The color of each pixel influenced by the structure of the image and provided by the standard observer, the digital camera and the two illuminants was then compared using an Euclidean distance between the two coordinates [26], as other color difference formulas are not recommended [38].

### 2.4. Error Distribution 

To estimate the frequency of errors for each illuminant, color space and color difference formula, the color difference between the colors obtained from the standard observer and the digital camera was estimated in each case. The frequency of the color difference was then estimated assuming 30 intervals (*K*) in each case, as predicted by Sturge’s rule [39] assuming the total number of pixels analyzed (*N* = 197,936,935 pixels) as the number of observations: $K=1+log_{e}\left(N\right)$. The distribution of the errors was then fitted with a Gaussian curve to extract the position of the center of the curve as the maximum of the errors estimated. The Gaussian curve was computed by:

(1) $$y=\;y_{0}+\frac{Ae^{\frac{-4\ln\left(2\right){\left(x-x_{c}\right)}^{2}}{w^{2}}}}{w\sqrt{\frac{\pi }{4\ln\left(2\right)}}},$$

with $y_{0}$ being the base of the curve, $x_{c}$ the center of the curve, *A* the area under the curve, and *w* the full width at half maximum (FWHM). $x_{c}$ was assumed to be the position of the most frequent error and taken as an indication of the magnitude of the chromatic difference between the colors computed assuming the standard observer and the digital camera. The higher this value, the higher the chromatic difference between them.

### 2.5. Number of Discernible Colors and Chromatic Volumes

To describe the overall chromatic differences between the natural scenes and the paintings, the number of discernible color and the color volume encompassing such colors were estimated.

The number of discernible colors was estimated in the CIELAB color space. The volume occupied by all the color of each image was segmented into unitary cubes. All cubes that had one or more colors inside were counted, assuming that all colors that were inside the same cube could not be discriminable. Counting the number of non-empty cubes provided a good estimation of the number of different colors in each image. Further details are described elsewhere [17,40]. To have an estimation of the chromatic diversity across images, the number of discernible colors was also estimated ignoring the L^*^ dimension. The area occupied by the colors was segmented into unitary squares and the number of discernible colors was estimated by counting the number of non-empty squares. It was assumed that all colors that were inside the same square could not be discriminable.

For each image and illuminant, the volume (or area if the L^*^ dimension was ignored) occupied by all the colors of a particular image was computed by estimating for each image the volume of a convex envelope that contained all the colors inside by using a routine from MATLAB (*convhull.m,* MathWorks, Natick, MA, USA). There was a need to estimate the color volume and the number of discernible colors, as the computation of the color volume will overestimate the gamut as will consider empty volumes. The use of the number of discernible colors will only consider actual existing colors. Further statistics were not considered in this work, as described elsewhere [41,42].

## 3. Results

### 3.1. Colors and Gamuts

**Figure 4:**
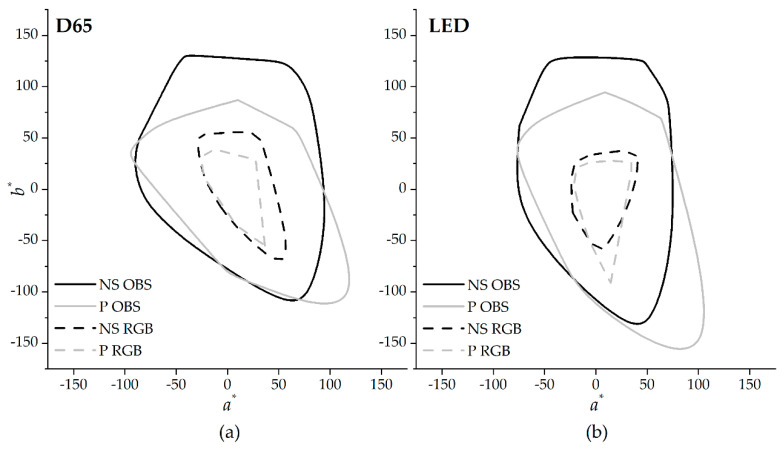
Color gamut in the CIE(a*,b*) color space generated by all the colors of paintings (light grey line) and natural scenes (dark line), for the CIE 1931 standard observer (OBS, full line) and the digital camera (RGB, dashed line). Data for the CIE D65 (panel (**a**)) and the LED (panel (**b**)) illuminants.

Figure 4 represents the estimated color gamut for natural scenes (black color) and paintings (light grey color), computed for the standard observer (OBS, full lines) and the digital camera (RGB, dashed lines) assuming the CIE D65 (panel (a)) and LED (panel (b)) illuminants.

Table 1 represents the descriptive information associated with the OBS and the RGB, for the CIE D65 and LED illuminants.

Both Figure 4 and Table 1 show that the color quantities are similar across illuminants, although the shape of the volumes or areas are slightly different. For example, the color volume occupied by all the colors of the natural scenes is similar for CIE D65 and LED illuminants, but the latter has a narrower and elongated shape along the a^*^ and the b^*^, respectively. The data for OBS is consistent with published computations for natural scenes [17] and paintings [42].

Table 2 summarizes the percentage variations found when comparing the volume and number of discernible colors (NODC) across all images (considering the colors of the natural scenes and paintings combined). The first and second rows present the data between OBS and RGB in percentage variation of the NODC and the color volume for the CIE D65 and LED illuminants. The third and fourth row present the data between CIE D65 and LED illuminants in percentage variation of the NODC and color volume for the standard observer OBS and the digital camera RGB. Both NODC and volume were estimated in the CIELAB color space and ignoring the L^*^ dimension (CIE(a^*^,b^*^)). It was found that the gamut provided by the RGB (dashed lines in Figure 4) is around 75% smaller than the one provided by the OBS (full lines in Figure 4) and that this value is independent of the illuminant tested. It was also found that for OBS, when comparing the NODC and volumes across illuminants, the variations are negligible, but when the RGB is considered, the value obtained with CIE D65 is around 10% higher than with the one obtained with LED.

The variations across illuminants seem to be independent for the OBS, but illuminant dependent if the RGB is considered. In both cases, as represented in panel A and B in Figure 4, the gamut available for the RGB is smaller than the one available for the OBS, and the shape and orientation is different. The change in size will limit the acquisition of colors with higher saturation, whereas the change in shape and orientation will impact the hues of the colors under acquisition. 

### 3.2. Color Differences

**Figure 5:**
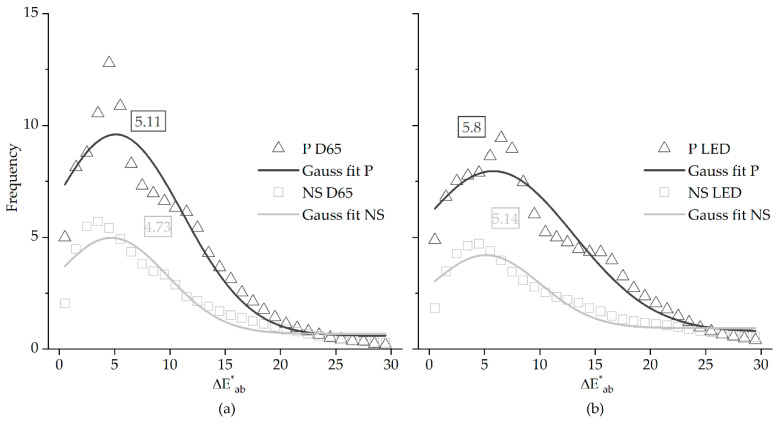
Color difference errors (ΔE^*^_ab_) in the CIELAB color space between the colors obtained for the CIE 1931 standard observer (OBS) and the digital camera (RGB), computed for paintings (open triangles) and natural scenes (open squares) under the CIE D65 (panel (**a**)) and the LED (panel (**b**)) illuminants. Full lines represent the best fit to the data of a Gaussian function. The numbers depicted correspond to the maximum color error (ΔE^*^_ab_) extracted from the function fitted.

Figure 5 represents the color errors between the CIE 1931 standard observer (OBS) and the digital camera (RGB). The color differences (ΔE^*^_ab_) were estimated in the CIELAB color space assuming the Euclidean distance between the two colors. Data in panel (a) was estimated considering the CIE D65 illuminant, paintings (open triangles and dark solid line) and natural scenes (open squares and light gray solid line—panel (b) as the same type of data considering the LED illuminant). Solid lines represent the Gaussian curve fit to the data as described in Equation (1). The number depicted inside the rectangles is the most frequent ΔE^*^_ab_ obtained from the position of the max of the curve fitted of 5.11 and 4.73, for paintings and natural scenes, respectively.

For the LED illuminant (panel (b)) it was found that the most frequent ΔE^*^_ab_ was 5.8 for images of paintings (open triangles and dark solid line) and 5.14 for images of natural scenes (open squares and light gray solid line), with the solid line representing the Gaussian curve fitted to the data.

Table 3 represents the same type of data as extracted from the position of the maximum of a Gaussian function fitted to the data, but considering the different color difference formulas, color spaces, illuminants for paintings, and natural scenes.

Data in Table 3 and the standard errors associated to the estimation of the maximum of each function suggests that the illuminant and the class of the image (art painting or natural scenes) does not influence the maximum frequency of the ΔE^*^_ab_. 

**Figure 6:**
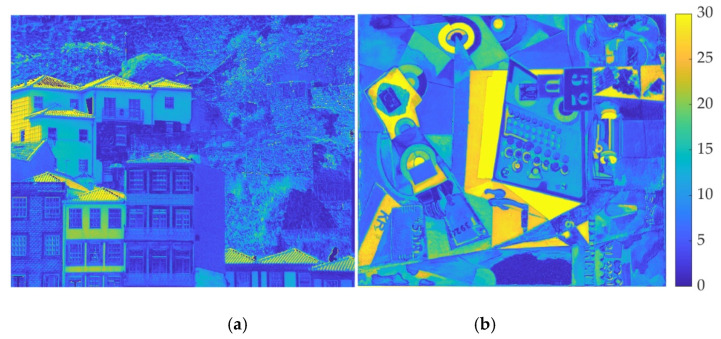
Color errors estimated between the RGB and the OBS in the CIELAB color space for a natural image (**a**) and a Portuguese painting (**b**) The original colors of the images are represented in Figure 2 Color bar represents the magnitude of the color errors (dark blue for no error and light yellow for a color error of 30).

As an illustration, Figure 6 shows the color errors for two scenes obtained when comparing the colors obtained for the OBS and the RGB, considering the CIELAB color space and the CIE D65 illuminant. On the left side (a) is a natural scene and on the right side (b) a painting from the Portuguese database. Both images were selected to be representative of the images analyzed and to contain some degree of chromatic saturation. The natural scene was also selected to contain natural and artificial colors. The color bar on the right represents the magnitude of the color errors, with dark blue being no color error, while light yellow represents a color error up to 30 units. Errors were estimated considering the chroma and the lightness of each image pixel. It can be seen that not all colors are affected in the same way and that there is some clustering around uniform surfaces characterized by a particular hue. This is the case for natural scenes and paintings.

**Figure 7:**
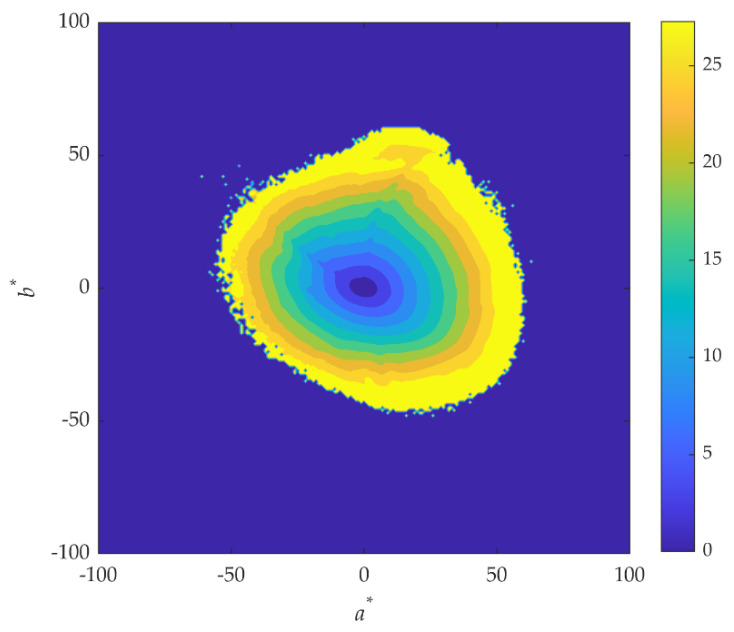
Color error distribution across the CIELAB color space for all natural scenes and paintings and CIE D65 illuminant. The right color bar represents the magnitude of the color errors.

Figure 7 represents the distribution of the color errors in the CIELAB color space assuming the CIE D65 Illuminant and all the colors in the database. The color bar on the right represents the magnitude of the color errors, with dark blue being no color error, while light yellow represents a color error up to 30 units. Errors were estimated considering the chroma and the lightness of each image pixel. The dark blue on the center of the figure represents less saturated colors that have smaller color error. As the saturation increases so the color errors increase, as the limited gamut of the RGB camera imposes a limit of colors that can be acquired without expected error. 

## 4. Discussion and Conclusions

Comparison between the actual colors and those produced by RGB cameras show that the most frequent error in the CIELAB was 5, for paintings and natural scenes. The value of the most frequent error was found to be similar across paintings and natural scenes regardless of the color space used, as represented in Table 3, and the differences in the absolute magnitude are due to the specificity of each color space. It is known that the texture of an image can change the accepted color difference tolerance [43]. Considering different types of texture, the tolerance for acceptance of color differences ΔE^*^_ab_ is, on average, higher than 8, while if the CIEDE2000 is considered in homogeneous samples, the tolerance is around 5 in the chroma alone, and around 3 in the hue and luminance [43]. If postcards reproductions of art paintings are considered, the postcard reproduction is considered to represent the painting even with a difference in chroma or color saturation (ΔC^*^_ab_) of about 8, depending on the illuminant in use [44]. The magnitude of ΔC^*^_ab_ is comparable to the magnitude of ΔE^*^_ab_, even if the former estimates color differences based on saturation while the latter estimates the color differences based on color coordinates. These tolerance values are higher than the values found here and higher than the threshold for chromatic difference detection in complex images of about 2.2 ΔE^*^_ab_ units [45], which may indicate that the RGB reproductions may be accepted by a real observer.

The distribution of errors across the color space shows that the errors are very small in the achromatic region and increase with saturation. This is expected given the gamut limitations of the RBG cameras. Nevertheless, the visual effects on most natural images may be subtle, as most of the natural colors have low saturation. 

Other studies using a limited sample of real colors estimated color differences of 2 to 4 Δ*E* in the CIELAB color space [2] after optimizing the computation of the RGB data with a training set similar to the colors to measure [4]. The data presented here seems to be higher (around 5 Δ*E*) but in comparison with the other data, no assumption was made in terms of data selection to perform the color difference test. In addition, no attempt was made to create a model different from the one already published [2] that would use training sets to improve the colorimetric outcome of digital cameras. When compared to other unconstrained acquisitions of colors under different illuminants [7,8,46], the results found here are better, mainly because the input data is exactly the same for the human observer and the digital camera. No variations exist in the acquisition setup when considering the two different acquisition methods, and a uniform illuminant distribution was assumed when considering both illuminants in both acquisition methods.

The optimization of the CIELAB color space for the CIE D65 illuminant is known. Nevertheless, using other illuminants in this color space to estimate variations and comparisons in color difference is acceptable, since no absolute values are to be estimated, but rather relative ones. 

The camera-specific settings were not considered in this analysis. It was assumed that the spectral stimulus was being received by the digital camera RGB sensor using the same optical components and camera settings that were in place at the time of the sensor spectral characterizations. Expanding the results found here to all possible camera settings and configurations should be done carefully, as each impacts the camera characterization differently [5]. Moreover, on the possible optimization of the color difference formulas to decrease the chromatic errors that were found [47], all color spaces and color difference formulas were used with standard parametric factors. The CIEDE2000 color difference formula accepts parametrizations, e.g., *kL* = 2, as suggested elsewhere [47]. The results in the present computations are, however, almost unaffected in relation to the default parametrization (±0.6 CIEDE2000 units in average, across all conditions). Possible improvements to the work flow of the RGB post processing pipeline or CCD array layout [48,49,50] were also not considered and tristimulus values were directly converted into sRGB values.

The use of complex images also increases the complexity of comparing colors, as one color is never seen isolated, but is always surrounded by colors. Color difference formulas tend to compare colors individually, independent of their surroundings. The iCAM06 color space was used to overcome this limitation, but it was found to have a limited impact on the overall result. 

Only images of natural scenes and paintings were considered in this work. Cameras can be used in extreme environments such as underwater, or in dentistry, [51,52] or in unmanned aircraft systems [53], but these extreme environments are outside of the scope of this paper.

Overall, the results presented here seems to indicate that the chromatic errors estimated are within the discrimination acceptance error when complex images are considered, but higher than the threshold assumed for such complex images.

## Figures and Tables

**Table 1:** Average color volume and correspondent number of discernible colors (NODC) estimated across paintings and natural scenes, for the CIE 1931 standard observer (OBS) and the digital camera (RGB) and the CIE D65 (D65) and LED (LED) illuminants considering the CIELAB color space. Also represented is the gamut area ignoring the L^*^ dimension and the correspondent number of discernible colors. Numbers in brackets represents the standard deviation. Data magnitude is (×10^3^).

		Paintings (×10^3^)		Natural Scenes (×10^3^)	
		D65	LED	D65	LED
**Volume**	**OBS**	160.9	162.0	439.2	441.5
		(± 126.4)	(± 126.3)	(± 284.5)	(± 284.8)
	**RGB**	25.0	22.9	69.6	61.0
		(± 18.2)	(± 17.6)	(± 45.42)	(± 39.2)
**NODC Volume**	**OBS**	43.4	43.4	92.2	94.4
		(± 32.5)	(± 32.4)	(± 48.2)	(± 49.5)
	**RGB**	10.6	9.5	25.7	22.8
		(± 7.2)	(± 6.9)	(± 14.9)	(±12.9)
**Area**	**OBS**	4.5	4.6	9.3	9.3
		(± 2.7)	(± 2.8)	(± 5.0)	(± 4.8)
	**RGB**	0.7	0.7	1.5	1.3
		(± 0.4)	(± 0.4)	(± 0.9)	(± 0.7)
**NODC Area**	**OBS**	2.8	2.8	5.1	5.2
		(± 1.7)	(± 1.7)	(± 2.4)	(± 2.4)
	**RGB**	0.6	0.5	1.1	1.0
		(± 0.3)	(± 0.3)	(± 0.5)	(± 0.5)

**Table 2:** Percentage variations of the data represented in the first two columns of Table 1, considering all the images analyzed, combining the data from paintings and natural scenes.

		NODC (%)		Color Volume (%)	
		CIELAB	CIE(a^*^,b^*^)	CIELAB	CIE(a^*^,b^*^)
**OBS vs. RGB**	D65	26.3	20.9	15.7	16.2
	LED	23.2	18.3	13.9	14.3
**D65 vs. LED**	OBS	101.3	100.8	100.9	100.2
	RGB	89.5	88.3	89.2	88.1

**Table 3:** Color errors for the color difference formulas and color spaces considered, for CIE D65 and LED illuminants for paintings and natural scenes. Error numbers represent the standard error associated to the fit.

	Paintings		Natural Scenes	
	D65	LED	D65	LED
**CIELAB**	5.1 (± 0.5)	5.8 (± 0.5)	4.7 (± 0.4)	5.1 (± 0.4)
**CIEDE2000**	5.7 (± 0.2)	6.2 (± 0.1)	5.9 (± 0.1)	6.1 (± 0.2)
**J_z_a_z_b_z_ (×10^−3^)**	34.5 (± 1.5)	35.2 (± 1.3)	74.0 (± 0.0)	73.3 (± 0.6)
**iCAM**	2.0 (± 0.1)	1.8 (± 0.1)	1.0 (± 0.2)	0.9 (± 0.1)

## Abbreviations

CCT	Correlated color temperature
CCD	Charge-coupled device
CIE	Commission International de l′Éclairage
OBS	Data related to tristimulus values and device independent
RGB	Data related to a digital camera and device dependent
sRGB	standard Red Green Blue color space
STD	Standard deviation
